# Measurement of patients’ acceptable symptom levels and priorities for symptom improvement in advanced prostate cancer

**DOI:** 10.1007/s00520-025-10299-x

**Published:** 2026-01-03

**Authors:** Stella Snyder, Ekin Secinti, Ellen F. Krueger, Nabil Adra, Roberto Pili, Nasser H. Hanna, Catherine E. Mosher

**Affiliations:** 1 Department of Psychology, Indiana University Indianapolis, 402 North Blackford Street, LD 124, Indianapolis, IN 46202 USA; 2 Department of Medicine, Indiana University School of Medicine, Indianapolis, IN USA; 3 Indiana University Melvin and Bren Simon Comprehensive Cancer Center, Indianapolis, IN USA; 4 Department of Medicine, Jacobs School of Medicine & Biomedical Sciences, University at Buffalo, Buffalo, NY USA

**Keywords:** Patient-centered outcomes, Patient-centered care, Advanced prostate cancer, Latent profile analysis, Symptom importance, Symptom severity

## Abstract

**Purpose:**

Limited research has evaluated the success criteria and priorities for symptom improvement of patients with cancer to inform patient-centered care. In this study, we adapted and tested a measure of these constructs, the Patient-Centered Outcomes Questionnaire (PCOQ), for patients with advanced prostate cancer. We compared acceptable symptom severity levels following symptom treatment across 10 symptoms and identified patient subgroups based on symptom importance.

**Methods:**

Patients with advanced prostate cancer (*N* = 99) participated in a one-time survey, which included a modified version of the PCOQ, standard symptom measures, and additional clinical characteristics.

**Results:**

The modified PCOQ demonstrated construct validity through its correlations with related theoretical constructs. There was a moderate correlation between symptom severity and importance. Acceptable symptom severity levels were generally low, with sexual dysfunction having a higher acceptable severity than most other symptoms. Three patient subgroups were identified: (1) those who rated all symptoms as low in importance (*n* = 43); (2) those who rated all symptoms as moderately important (*n* = 33); and (3) those who rated all symptoms as highly important (*n* = 18). Subgroups were associated with functional status, fatigue, sleep problems, pain, and emotional distress.

**Conclusion:**

The modified PCOQ demonstrated preliminary evidence of construct validity. Patients generally considered low symptom severity to be acceptable, with variations across symptoms. Results suggest that symptom severity and importance are related but distinct aspects of the symptom experience in advanced prostate cancer. Patients’ diverse priorities for symptom improvement point to the need for individualized treatment plans.

**Supplementary Information:**

The online version contains supplementary material available at 10.1007/s00520-025-10299-x.

## Introduction

Patient-centered care emphasizes shared decision-making and providing care that is responsive to individual needs and values [1, 2]. This approach can lead to improved patient satisfaction, health outcomes, and treatment adherence [3–5]. Within supportive cancer care, shared decision-making helps align evidence-based treatments with patients’ expectations and priorities and empowers patients to actively manage their symptoms [4, 6–8]. Researchers have rarely assessed patients’ criteria for successful symptom treatment and their priorities for symptom improvement in cancer care. A deeper understanding of patients' views on acceptable levels of symptom severity and the importance of treating certain symptoms can strengthen patient-provider communication and facilitate the development of care plans targeting specific needs [9].

The Patient-Centered Outcomes Questionnaire (PCOQ) has been used to define treatment success and priorities for symptom improvement from the patients’ perspective [2]. Primarily used within chronic pain and Parkinson’s disease research, this measure has revealed a significant gap between patient expectations for treatment success and the actual effectiveness of existing therapies, underscoring the need for care that better aligns with patient preferences [2, 3, 5, 10–12]. Within these populations, the PCOQ has also identified patient clusters based on their ratings of the importance of improving pain, fatigue, emotional distress, and daily activities [2, 12]. This differentiation has the potential to enhance personalized care approaches.

Modified versions of the PCOQ have been used with two cancer patient groups [5, 13, 14]. First, the PCOQ was revised to focus on 10 common symptoms in metastatic breast cancer [14]. Patients indicated that fatigue, cognitive problems, and sleep problems would require the greatest reductions to consider symptom treatment successful (range = 43–49% reductions). Additionally, three distinct patient clusters based on ratings of the importance of symptom improvement were found: (1) those who rated cognitive problems, fatigue, and sleep problems as highly important, (2) those who rated pain as highly important, and (3) those who rated all symptoms as highly important. The PCOQ was also revised to focus on eight common symptoms in advanced lung cancer [13]. In general, levels of acceptable symptom severity were low and only small reductions from usual symptom severity were needed to reach acceptable levels. Four patient clusters based on ratings of the importance of symptom improvement were found: (1) those who considered all symptoms as minimally important; (2) those who rated bronchial symptoms and sleep issues as low in importance and other symptoms as moderately important; (3) those who rated nausea and emotional distress as low in importance and other symptoms as moderately important; and (4) those who considered all symptoms to be highly important. Across studies, results demonstrate diversity with respect to patient success criteria and priorities for symptom treatment.

Outcomes specific to breast and lung cancers may not generalize across all advanced cancers. Patients with advanced prostate cancer often present with distinctive symptoms that significantly affect their quality of life, such as urinary problems and sexual dysfunction [15, 16]. Limited prostate cancer research addresses patient perspectives on success criteria and symptom improvement priorities [17–19]. This research has primarily assessed the importance of urinary incontinence and sexual impotence in patients with early-stage prostate cancer [17–19].

This study adapted the PCOQ for patients with advanced prostate cancer, focusing on 10 prevalent symptoms. For each symptom, we evaluated typical symptom severity, acceptable symptom severity after symptom treatment, and the importance of improving the symptom. The primary aim of this study was to evaluate the construct validity of this modified PCOQ. The Dodd Symptom Management Model suggests that symptom experiences are associated with demographics, medical factors, and outcomes such as functional status and quality of life [20]. Potential correlates of the PCOQ were selected based on this model. We hypothesized that symptom severity ratings on the PCOQ would be correlated with established symptom assessments, medical comorbidities, functional status, quality of life, and the importance of symptom improvement. As secondary aims, this study (2) compared acceptable severity levels after symptom treatment across the 10 symptoms, (3) identified patient subgroups based on symptom improvement priorities, and (4) explored demographic and clinical correlates of patient subgroups based on symptom improvement priorities.

## Methods

### Participants and procedures

After Indiana University (IU) Institutional Review Board approval, participants were recruited from the IU Health University Hospital and IU Health cancer registry to participate in a one-time survey between March and August 2019. Eligibility criteria included: 1) ≥ 3 weeks post-diagnosis of stage IV prostate cancer; 2) ≥ 18 years old; 3) English fluency; and 4) no evidence of severe cognitive impairment (< 3 errors on a 6-item cognitive screener) [21]. Recruitment materials were sent to potential participants with information for opting out of the study. Research assistants contacted patients who did not opt out, administered the cognitive screener, and obtained consent. Online or paper surveys were sent to consenting participants based on their preference. Reminder calls or automated emails were sent as reminders to complete the survey. Participants received a $25 gift card upon survey receipt.

### Measures

#### Demographics and medical factors

Participants’ age and cancer-related information were collected from medical records. Other demographics were self-reported. Patients reported whether they were diagnosed or treated for eight common medical conditions in the past three years [22]. Using an author-constructed checklist, patients also reported their treatments in the past 3 years (i.e., over-the-counter or prescribed medication, oxygen, psychotherapy/counseling, or other treatments) for each of the 10 symptoms in the modified PCOQ. Functional status was assessed using one item from the Patient-Generated Subjective Global Assessment (PG-SGA) [23].

#### Modified PCOQ

The original PCOQ was developed for people with chronic pain [2] and has demonstrated evidence of reliability and concurrent validity with pain, disability, and distress measures [3]. Based on a review of the literature and feedback on the PCOQ from prior cognitive interviews with patients with advanced cancer [13], we modified the PCOQ to focus on 10 common symptoms in patients with prostate cancer in three sections. For each symptom, patients first rated their usual symptom severity during the past week on an 11-point scale (0 = none, 10 = worst imaginable). Participants with a usual symptom severity greater than zero were then asked: “What level of [symptom] would be acceptable to you if you were to receive treatment for [symptom]?” on an 11-point scale (0 = none, 10 = worst imaginable) and, “How important is it for you to see improvement in your level of [symptom]?” on an 11-point scale (0 = not at all important, 10 = most important).

#### Symptom severity

The 3-item Patient-Reported Outcomes Measurement Information System (PROMIS) measure was used to assess pain intensity in the past week on a 5-point scale (1 = no pain/had no pain, 5 = very severe) [24]. Four-item PROMIS measures were used to assess anxiety, depressive symptoms, fatigue, and sleep disturbance in the past week on a 5-point scale (1 = never/not at all, 5 = very much). For all PROMIS measures, T-scores anchored to the U.S. general population norms were computed (Mean = 50, SD = 10). Memorial Symptom Assessment Scale (MSAS) subscales were used to assess urination problems, diarrhea, constipation, nausea, lack of appetite, and sexual dysfunction in the past week [25]. For MSAS measures, participants rated the frequency (1 = rarely, 4 = almost constantly), severity (1 = slight, 4 = very severe), and level of distress (0 = not at all, 4 = very much) associated with each symptom.

#### Quality of life

One item from the McGill Quality of Life Questionnaire was used to assess global quality of life over the past two days on an 11-point scale (0 = very bad, 10 = excellent) [26].

### Data analysis

Descriptive statistics were computed using SPSS v.25.0. For aim 1, the construct validity of the modified PCOQ was assessed through correlations between PCOQ symptom severity ratings, PROMIS and MSAS measures of the same symptoms, and theoretically related constructs (i.e., medical comorbidities, functional status, and quality of life).

For aim 2, acceptable severity levels following symptom treatment across the 10 symptoms were compared for patients who endorsed at least one symptom (i.e., usual severity rating ≥ 1 on a 0 to 10 scale) using linear mixed modeling in SPSS. Linear mixed modeling was chosen to allow an unbalanced design, accommodating participants who only provided acceptable severity levels for certain symptoms. In the model, acceptable symptom severity was the outcome, and symptom type (e.g., anxiety, pain) was the within-subjects predictor variable.

For aim 3, patient subgroups based on importance ratings for each of the 10 symptoms were identified using latent profile analysis (LPA) in Mplus v.8 [27]. To identify the best fitting model, five models were estimated, and subgroups were added iteratively. Based on the following fit indices, acceptable model fit was determined as: 1) lower values of Akaike Information Criterion (AIC), Bayesian Information Criterion (BIC), and sample size adjusted Bayesian Information Criterion (ssBIC), 2) statistically significant value for the bootstrap likelihood ratio test (BLRT), 3) entropy > 0.80, and 4) an interpretable model solution [28–31].

For aim 4, potential correlates of the patient subgroups were explored individually using multinomial logistic regressions with Vermunt’s 3-step approach in Mplus [32]. Examined correlates included usual severity of the 10 symptoms on the modified PCOQ, demographics (i.e., age, marital status, education, employment status, and income), and medical characteristics (i.e., number of medical comorbidities, functional status, time since the advanced cancer diagnosis, cancer treatment history, symptom treatment history, and quality of life). Given the number of analyses, *p*-values < 0.01 were considered statistically significant.

## Results

The study flow is found in Online Resource Fig. 1. Of the 197 patients who were sent recruitment mailings, 160 (81%) were reached via phone, 33 (17%) could not be reached, and 4 (2%) were deceased. Of those reached, 40 (25%) declined to participate mostly due to lack of interest or time, and 120 (75%) completed the eligibility screening. All eligible patients (*n* = 112) consented to participate, and most consenting patients (*n* = 99, 88%) completed the survey.

**Fig. 1:**
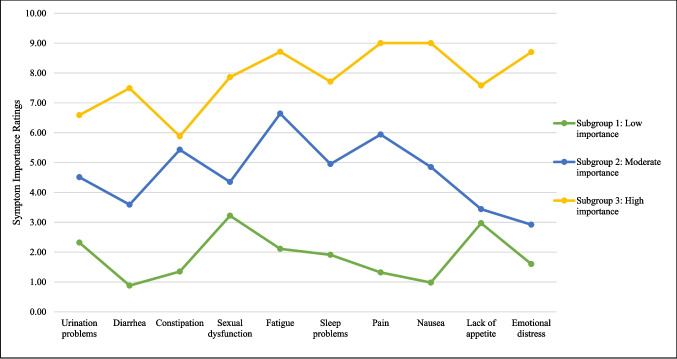
Patient subgroups’ estimated marginal mean importance ratings on the Patient-Centered Outcomes Questionnaire (PCOQ). *N* = 94

Participants were mostly non-Hispanic White (88%), with an average age of 68.49 years (*SD* = 9.01) (Table 1). Participants were, on average, 3.41 years (*SD* = 2.83) from their advanced prostate cancer diagnosis, and 56.57% were currently receiving hormonal therapy.

**Table 1:** Participant demographic and medical characteristics (*N* = 99)

Characteristic	Statistic
Age	
Mean (S.D.)	68.49 (9.01)
Range	42–87
Race and Ethnicity, no. (%)	
Non-Hispanic White	87 (87.88%)
African American/Black	9 (9.09%)
Other^*a*^	3 (3.03%)
Married or Living with a Partner, no. (%)	83 (83.84%)
Employed, no. (%)	40 (40.40%)
Level of Education, no. (%)	
No college	21 (21.21%)
Some college	22 (22.22%)
Graduated college/graduate school	54 (54.54%)
Household Income,^*b*^ no. (%)	
$0—$30,999	9 (9.09%)
$31,000—$50,999	21 (21.21%)
$51,000—$99,999	29 (29.29%)
$100,000 or more	36 (36.36%)
Years since Advanced or Extensive Stage Diagnosis	
Mean (S.D.)	3.41 (2.83)
Range	0.10—15.29
Cancer Treatment History,^*c*^ no. (%)	
Surgery	79 (79.80%)
Chemotherapy	36 (36.36%)
Radiation	45 (45.45%)
Targeted therapy	2 (2.02%)
Immunotherapy	14 (14.14%)
Hormone therapy	82 (82.83%)
Current Cancer Treatment,^*d*^ no. (%)	
Chemotherapy	12 (12.12%)
Radiation	1 (1.01%)
Targeted therapy	0 (0.00%)
Immunotherapy	4 (4.04%)
Hormone therapy	56 (56.57%)
Symptom Treatment History,^*e*^ no. (%)	
Urination problems	17 (17.17%)
Diarrhea	9 (9.09%)
Constipation	15 (15.15%)
Sexual dysfunction	8 (8.08%)
Fatigue	10 (10.10%)
Sleep problems	15 (15.15%)
Pain	23 (23.23%)
Nausea	3 (3.03%)
Lack of appetite	1 (1.01%)
Emotional distress	5 (5.05%)
Medical Comorbidities	
Mean (S.D.)	1.08 (1.04)
Range	0–5

^*a*^Multi-racial, Asian American, Native American, Hispanic, and other

^*b*^Median household income in Indiana was $57,603 according to U.S. Census 2019 data. Reference: United States Census Bureau (2020) Indiana 2019. https://data.census.gov/cedsci/profile?g=0400000US18. Accessed 2 February 2024

^*c*^Treatment any time before study completion

^*d*^Treatment ≤ 4 weeks before study completion

^*e*^Treatment in the past 3 months for a particular symptom

PCOQ descriptive statistics are reported in Table 2. On average, patients reported experiencing 4.72 symptoms (*SD* = 2.37) during the past week. As evidence of the construct validity of the PCOQ (aim 1), the usual severity of all symptoms on the PCOQ were strongly correlated with standard assessments of the same symptoms, including the PROMIS and MSAS, *rs*(97–99) = 0.56–0.89, *ps* < 0.01 (Table 3). Only lack of appetite was significantly correlated with medical comorbidities, *r*(99) = 0.26, *p* < 0.01, whereas other symptoms showed small, non-significant correlations with medical comorbidities. More severe constipation, fatigue, pain, nausea, lack of appetite, and emotional distress were correlated with worse functional status, *rs*(97–99) = 0.30–0.54, *ps* < 0.01. More severe urination problems, fatigue, sleep problems, pain, nausea, and emotional distress were correlated with worse quality of life, *rs*(97–99) = −0.50 – −0.30, *ps* < 0.01. Moderate, positive correlations were found between the severity and importance of all symptoms, *rs*(13–82) = 0.56–0.78, *ps* < 0.01, except for sexual dysfunction and lack of appetite. Although severity and importance were also positively correlated for sexual dysfunction and lack of appetite, results fell short of statistical significance, *rs*(22–60) = 0.27–0.48, *ps* < 0.05.

**Table 2:** Descriptive statistics for PCOQ constructs

	Usual Severity				Acceptable Severity				Importance of Improvement			
Symptom	*n*	Mean	S.D	Range	*n*	Mean	S.D	Range	*n*	Mean	S.D	Range
Urination problems	98	2.36	2.81	0–10	59	2.59	1.78	0–6	59	4.00	3.30	0–10
Diarrhea	97	0.62	1.58	0–8	21	1.14	0.91	0–3	21	3.67	3.34	0–10
Constipation	98	1.06	2.15	0–10	26	2.19	1.83	0–6	26	4.23	3.31	0–10
Sexual dysfunction	97	4.20	4.17	0–10	59	3.66	2.68	0–10	60	4.55	3.70	0–10
Fatigue	98	3.62	2.54	0–10	81	2.51	1.74	0–8	82	5.05	3.21	0–10
Sleep problems	99	2.93	2.70	0–10	72	2.51	1.96	0–10	74	4.15	3.01	0–10
Pain	98	2.05	2.56	0–10	52	2.44	2.25	0–10	53	4.72	3.37	0–10
Nausea	98	0.40	1.25	0–7	14	1.71	1.59	0–6	13	4.46	3.69	0–10
Lack of appetite	99	0.85	1.94	0–10	22	3.41	2.32	0–10	22	4.82	3.26	0–10
Emotional distress	99	1.35	2.05	0–8	45	2.44	1.66	0–7	46	3.65	3.11	0–10

*PCOQ* Patient-Centered Outcomes Questionnaire

**Table 3:** Correlations between PCOQ symptom severity and hypothesized variables

	Standardized Symptom Severity ^*a*^		Medical Comorbidities ^*b*^		Functional Status ^*c*^		Quality of Life ^*d*^		Symptom Importance ^*e*^	
	*r*	*p*-value	*r*	*p*-value	*r*	*p*-value	*r*	*p*-value	*r*	*p*-value
Urination problems	0.72	< 0.001*	0.17	0.088	0.13	0.203	−0.35	< 0.001*	0.59	< 0.001*
Diarrhea	0.88	< 0.001*	0.08	0.460	0.16	0.125	−0.11	0.302	0.57	0.001*
Constipation	0.89	< 0.001*	0.20	0.054	0.31	0.002*	−0.25	0.012	0.73	< 0.001*
Sexual dysfunction	0.66	< 0.001*	0.17	0.092	0.12	0.257	−0.16	0.116	0.27	0.034
Fatigue	0.80	< 0.001*	0.03	0.799	0.54	< 0.001*	−0.50	< 0.001*	0.56	< 0.001*
Sleep problems	0.71	< 0.001*	0.09	0.398	0.26	0.010	−0.36	< 0.001*	0.60	< 0.001*
Pain	0.81	< 0.001*	0.24	0.018	0.53	< 0.001*	−0.42	< 0.001*	0.69	< 0.001*
Nausea	0.64	< 0.001*	0.25	0.012	0.30	0.003*	−0.30	0.003*	0.78	0.002*
Lack of appetite	0.83	< 0.001*	0.26	0.008*	0.39	< 0.001*	−0.23	0.022	0.48	0.023
Emotional distress	0.57, 0.55^*f*^	< 0.001*	0.00	0.969	0.30	0.003*	−0.45	< 0.001*	0.65	< 0.001*

Pairwise correlations. *PCOQ* Patient-Centered Outcomes Questionnaire

^*^p < 0.01

^*a*^ Memorial Symptom Assessment Scale (MSAS) measures were used for urination problems, diarrhea, constipation, sexual dysfunction, nausea, and lack of appetite, and Patient-Reported Outcomes Measurement Information System (PROMIS) measures were used for fatigue, sleep problems, pain, and emotional distress (i.e., anxiety and depression), *n*s = 96–99

^*b*^* n*s = 97–99

^*c*^* n*s = 97–99

^*d*^* n*s = 97–99

^*e*^* n*s = 13–82

^*f*^ Separate correlations were computed between PCOQ emotional distress and PROMIS anxiety and depression measures, *r*s = 0.57, 0.55, respectively

Among participants experiencing the relevant symptom, on average, a small reduction from usual severity (1.07 to 3.32 on a 0–10 scale) was considered acceptable. Regarding aim 2, estimated marginal means obtained from the linear mixed model analysis indicated that the lowest acceptable severity was 1.29 for diarrhea and the highest acceptable severity was 3.71 for sexual dysfunction (Table 4). Comparisons of acceptable severity across symptoms indicated that sexual dysfunction had significantly higher acceptability than all other symptoms except for urination problems and lack of appetite, *ps* < 0.01.

**Table 4:** Acceptable levels of symptom severity using linear mixed modeling (*n* = 97)

	Estimated Marginal Mean	S.E
Symptoms		
Urination problems	2.68	0.25
Diarrhea	1.29	0.39
Constipation	1.99	0.35
Sexual dysfunction	3.71	0.25
Fatigue	2.51	0.22
Sleep problems	2.51	0.23
Pain	2.37	0.26
Nausea	1.55	0.47
Lack of appetite	2.99	0.38
Emotional distress	2.42	0.28

Each symptom was rated on a 0 to 10 scale

Regarding aim 3, patient subgroups based on symptom improvement priorities were identified through estimation of five latent profile models (Online Resource 1). The 3-subgroup model was chosen because it yielded the lowest BIC value, provided a better fit than the 4-subgroup model based on the BLRT, and was the most conceptually meaningful classification of patients. Accordingly, subgroup 1 rated all symptoms as low in importance (*n* = 43, 43%); subgroup 2 rated all symptoms as moderate in importance (*n* = 33, 33%); and subgroup 3 rated all symptoms as high in importance (*n* = 18, 18%) (Fig. 1, Table 5).

**Table 5:** Descriptive statistics for subgroups based on symptom importance

	Subgroup 1			Subgroup 2			Subgroup 3		
Symptom Importance	Estimated Marginal Means	S.E	Range	Estimated Marginal Means	S.E	Range	Estimated Marginal Means	S.E	Range
Urination problems	2.32	0.58	0–10	4.51	0.66	0–9	6.59	0.82	0–10
Diarrhea	0.88	0.66	0–10	3.59	0.72	0–9	7.49	0.77	0–6
Constipation	1.35	0.87	0–5	5.43	0.97	0–9	5.88	0.89	0–10
Sexual dysfunction	3.22	0.67	0–10	4.35	0.76	0–10	7.86	1.02	0–10
Fatigue	2.11	0.29	0–10	6.64	0.34	0–10	8.71	0.44	0–10
Sleep problems	1.91	0.36	0–10	4.95	0.42	0–9	7.71	0.56	0–10
Pain	1.32	0.33	0–10	5.94	0.35	0–10	9.00	0.45	0–10
Nausea	0.98	1.36	0–3	4.85	0.92	0–9	9.00	1.73	0–10
Lack of appetite	2.97	0.96	0–8	3.44	0.90	0–7	7.58	0.85	0–10
Emotional distress	1.60	0.36	0–9	2.92	0.36	0–10	8.70	0.46	0–10
Differing Variables	Mean	SD	Range	Mean	SD	Range	Mean	SD	Range
Functional status	0.67	0.64	0–3	0.73	0.91	0–3	1.00	0.91	0–3
Fatigue	3.62	2.52	0–9	3.39	2.52	0–8	4.00	2.72	0–10
Sleep problems	2.86	2.61	0–9	2.70	2.62	0–8	2.72	2.24	0–7
Pain	2.02	2.33	0–8	1.91	2.59	0–8	2.17	2.64	0–7
Emotional distress	1.30	2.04	0–8	1.06	1.78	0–7	1.83	2.62	0–8
	*n*	(%)		*n*	(%)		*n*	(%)	
History of pain treatment	9	20.93%		9	27.27%		5	27.78%	

*Subgroup 1* Low Symptom Importance, *n* = 43; *Subgroup 2* Moderate Symptom Importance, *n* = 33; *Subgroup 3* High Symptom Importance, *n* = 18

Regarding aim 4, differences between subgroups on demographic and clinical characteristics were explored through multinomial logistic regressions using Vermunt’s 3-step approach with subgroup 1 as the reference group. Worse functional status (OR = 6.05), higher levels of fatigue (OR = 1.61), sleep problems (OR = 1.50), pain (OR = 1.64), and emotional distress (OR = 1.64), and a history of pain treatment (OR = 13.57) were associated with a greater likelihood of being in subgroup 3 than subgroup 1 (Online Resource 2). Similarly, worse functional status (OR = 6.52), higher levels of fatigue (OR = 1.57), sleep problems (OR = 1.43), pain (OR = 1.56), and a history of pain treatment (OR = 9.85) were associated with a greater likelihood of being in subgroup 2 than subgroup 1.

## Discussion

The modified PCOQ showed evidence of construct validity in patients with advanced prostate cancer. This measure provides thresholds for acceptable symptom severity and priorities for symptom improvement—concepts not addressed by current assessments for this population. The correlations between the PCOQ and related theoretical constructs were mostly aligned with the Dodd Symptom Management Model [20]. On average, patients experiencing symptoms considered a minor reduction in symptom severity acceptable. Acceptable symptom levels were significantly higher for sexual dysfunction than most other symptoms. Patient priorities for symptom treatment varied widely and were correlated with certain symptom levels and other clinical characteristics.

Results on the PCOQ’s construct validity were largely consistent with our hypotheses. The severity of all symptoms on the PCOQ was strongly correlated with standard measures of the same symptoms. For the majority of symptoms, greater severity was correlated with worse functional status and quality of life, providing further evidence of the PCOQ’s validity. Among the symptoms, only lack of appetite, prevalent in many clinical conditions [33–35], showed a significant correlation with the number of medical comorbidities. Additionally, moderate, positive correlations were found between symptom severity and importance for all symptoms except for sexual dysfunction and lack of appetite, suggesting that symptom severity and importance are related but distinct concepts. Previous research in chronic pain and cancer has found similar moderate associations between symptom severity and patient subgroups based on symptom importance [2, 5, 11, 13, 14, 36]. Patients may rate a mild symptom as highly important (e.g., due to functional impact or personal values) or a severe symptom as less important.

Potential explanations for the moderate correlation between symptom severity and importance have rarely been assessed. In one qualitative study, patients with advanced lung cancer provided varied reasons for symptom importance ratings that extended beyond symptom severity [13]. For example, some patients reported that these ratings reflected their ability to tolerate the symptom or the extent to which the symptom interfered with daily activities. Others stated that their low symptom importance ratings were due to prioritizing survival over symptom management, suggesting trade-offs between symptom burden and perceived treatment benefit. Similarly, a qualitative study in metastatic breast cancer found that perceptions of symptom importance were not solely based on symptom severity, but factors such as activity limitations, health concerns, and relationship impacts [37].

Patients generally preferred low symptom severity levels across all symptoms and considered minor reductions in symptom severity acceptable, although acceptability varied across symptoms. The acceptable severity levels align with success criteria for symptom improvement in patients with chronic pain and metastatic breast and lung cancer [2, 10, 13, 14, 36]. The lowest acceptable severity ratings were found for diarrhea and nausea, emphasizing their importance as intervention targets. A previous study of patients with prostate cancer identified diarrhea as a more troubling side effect than urinary symptoms, underscoring its significance [38]. However, in this study, patients, on average, had very mild diarrhea and nausea. Conversely, sexual dysfunction, a prevalent side effect of prostate cancer therapies, had a significantly higher acceptable severity level than most symptoms. This side effect may be addressed in patient education [39], which could help facilitate adaptive coping strategies. Additionally, reduced sexual desire, a frequent side effect of treatment [40, 41], may lead to greater tolerance of erectile dysfunction. The higher acceptability rating may reflect resignation or adaptation. Indeed, qualitative findings suggest some men with prostate cancer find satisfaction in non-erection-dependent intimacy despite persistent dysfunction [42]. The severity and importance of sexual dysfunction were not significantly correlated in this study, consistent with its higher acceptable severity level. While the term "sexual dysfunction" does not differentiate between specific sexual domains (e.g., erectile function, libido), this broad wording was used to provide a global patient-centered indicator of sexual concerns.

Fatigue severity was the most distant from its acceptable level, aligning with research that identifies fatigue as the most troublesome symptom for patients with advanced prostate cancer [43]. However, in this study, fatigue did not require substantial reduction to be considered manageable.

Three distinct subgroups of patients were identified based on the importance of seeing symptom improvement: those who rated all symptoms as low, moderate, or high in importance. This classification illustrates the variability in patient attitudes towards symptom management. Studies in advanced breast and lung cancer also identified one patient subgroup that rated all symptoms as highly important [13, 14]. Differences in the remaining patient subgroups across studies likely reflect variations in the examined symptoms and sample characteristics.

In the current study, patients who rated all symptoms as moderately or highly important were more likely to have greater physical symptoms (e.g., pain, fatigue), a history of pain treatment, and worse functional status than patients who rated all symptoms as low in importance. In addition, patients who rated all symptoms as highly important showed higher levels of emotional distress than patients who rated all symptoms as low in importance. The moderate and high importance subgroups had several common correlates (e.g., worse functional status, greater fatigue, pain, and sleep problems), suggesting a continuum of symptom burden rather than distinct profiles. These common characteristics between subgroups may help providers identify patients who are more likely to prioritize symptom management. Our findings align with the Dodd Symptom Management Model, which posits correlations between health-related characteristics and symptom experiences [20]. Other findings in advanced breast and lung cancer and chronic pain were largely inconsistent with this model; few demographic and clinical correlates of patient subgroups based on symptom importance were found [2, 10, 13, 14, 36]. Findings suggest that for patients with advanced prostate cancer, symptom importance ratings may more closely parallel other aspects of their functioning and clinical care as compared to patients with other advanced solid tumors or chronic pain.

Limitations of the current study should be noted. This study was conducted at a single academic medical center in the midwestern United States and primarily enrolled non-Hispanic White participants. The sample’s homogeneity limits generalizability, as cultural beliefs, healthcare access, and social determinants of health may affect symptom priorities and definitions of acceptable symptom severity. In addition, the relatively small sample size may have reduced statistical power for detecting significant associations, and the cross-sectional design did not allow for the evaluation of test–retest reliability and longitudinal changes. Finally, we did not collect standardized indicators of treatment response (e.g., PSA trends) or detailed regimen data (e.g., specific agents/doses or orchiectomy status). These factors warrant study in relation to patient-centered symptom outcomes.

Our findings highlight the need for personalized symptom management for patients with advanced prostate cancer. This approach would incorporate patient criteria for symptom treatment success and treatment priorities into the shared decision-making process. Specifically, patients in our study often required low symptom severity to view treatment as satisfactory, underscoring the need for tailored patient-provider discussions about expected treatment outcomes. Additionally, given the varied priorities among patients for symptom improvement, understanding individual treatment goals would inform patient-centered care. Although the modified PCOQ was developed for research use, future work may develop a brief screener based on the PCOQ for clinical application. For example, clinicians could incorporate two brief questions into routine assessment: “Which symptom is most important to treat?” and “What level of this symptom would be acceptable to you?” These items reflect the PCOQ’s core constructs and may support tailored symptom discussions in practice. Finally, it is important for providers to consider the interconnectedness of symptoms and discuss potential side effects of treatments to align with patients’ symptom management priorities.

There are several important directions for future research. The psychometric properties of our modified PCOQ should be examined in larger advanced prostate cancer samples that are fully representative of the population. Additionally, researchers could modify and validate the PCOQ for other advanced cancer populations. Longitudinal studies could assess changes in acceptable symptom levels and priorities for symptom improvement over the cancer trajectory. Examining how symptom priorities shift across treatment phases, life transitions, and goals-of-care discussions will inform personalized symptom management. Future studies could also examine how objective and perceived disease control relates to symptom acceptability. Additionally, studies could assess distinct sexual domains (e.g., libido vs. erectile function) to evaluate whether acceptability and priorities differ across these concerns. Finally, pragmatic intervention trials targeting symptom clusters could assess patient priorities for symptom improvement to guide the steps in their care. This research will lead to patient-centered approaches to enhancing quality of life and functioning in advanced cancer.

## Supplementary Information

Below is the link to the electronic supplementary material.

Supplementary file1 (DOCX 15 KB)

Supplementary file2 (DOCX 18 KB)

Supplementary file3 (DOCX 31 KB)

## Data Availability

The datasets generated during and/or analyzed during the current study are available from the corresponding author on reasonable request.
