# Supplementary material for: Measurement of patients’ acceptable symptom levels and priorities for symptom improvement in advanced prostate cancer
Source: Support Care Cancer. 2026 Jan 3;34(1):63. doi: 10.1007/s00520-025-10299-x (PMC12764673; doi:10.1007/s00520-025-10299-x)
Supplement: Supplementary file 1 — Supplementary file1 (DOCX 15 KB) [file 520_2025_10299_MOESM1_ESM.docx]

| **Online Resource 1** Measures of Model Fit for Latent Profile Analysis | | | | | | |
| --- | --- | --- | --- | --- | --- | --- |
| Subgroups | LL | AIC | BIC | ssBIC | E | BLRT |
| 1 | -1184.29 | 2408.58 | 2459.44 | 2396.30 | N/A | N/A |
| 2 | -1096.65 | 2255.30 | 2334.14 | 2236.28 | 0.86 | *p* < .01 |
| 3 | -1061.60 | 2207.19 | 2314.01 | 2181.42 | 0.88 | *p* < .01 |
| 4 | -1043.31 | 2192.63 | 2327.42 | 2160.10 | 0.87 | *p* = .03 |
| 5 | -1023.74 | 2175.48 | 2338.25 | 2136.21 | 0.89 | *p* = .07 |
| LL = Log Likelihood. AIC = Akaike Information Criterion. BIC = Bayesian Information Criterion. ssBIC = Sample Sized Adjusted Bayesian Information Criterion. E = Entropy. BLRT = Bootstrap Likelihood Ratio Test. | | | | | | |
