# Supplementary material for: Measurement of patients’ acceptable symptom levels and priorities for symptom improvement in advanced prostate cancer
Source: Support Care Cancer. 2026 Jan 3;34(1):63. doi: 10.1007/s00520-025-10299-x (PMC12764673; doi:10.1007/s00520-025-10299-x)
Supplement: Supplementary file 2 — Supplementary file2 (DOCX 18 KB) [file 520_2025_10299_MOESM2_ESM.docx]

| **Online Resource 2** Multinomial logistic regression results comparing patient subgroups based on symptom importance | | | | | | | |
| --- | --- | --- | --- | --- | --- | --- | --- |
|  | Subgroup 1 vs. 2 | | |  | Subgroup 1 vs. 3 | | |
| Differing Variables | OR | 99% CI | *p*-value |  | OR | 99% CI | *p*-value |
| Functional Status | 6.52 | 1.52, 28.09 | 0.001 |  | 6.05 | 1.39, 26.42 | 0.002 |
| Fatigue | 1.57 | 1.10, 2.25 | 0.001 |  | 1.61 | 1.09, 2.40 | 0.002 |
| Sleep problems | 1.43 | 1.05, 1.94 | 0.002 |  | 1.50 | 1.06, 2.10 | 0.003 |
| Pain | 1.56 | 1.10, 2.22 | 0.001 |  | 1.64 | 1.13, 2.40 | 0.001 |
| Emotional distress | 1.32 | 0.85, 2.04 | 0.103 |  | 1.64 | 1.05, 2.56 | 0.004 |
| History of pain treatment | 9.85 | 1.01, 96.55 | 0.010 |  | 13.57 | 1.31, 140.75 | 0.004 |
| OR = Odds Ratio. CI = Confidence Interval. *Subgroup 1* Low Symptom Importance, *n* = 43; *Subgroup 2* Moderate Symptom Importance, *n* = 33; *Subgroup 3* High Symptom Importance, *n* = 18. | | | | | | | |
