# Supplementary material for: Measurement of patients’ acceptable symptom levels and priorities for symptom improvement in advanced prostate cancer
Source: Support Care Cancer. 2026 Jan 3;34(1):63. doi: 10.1007/s00520-025-10299-x (PMC12764673; doi:10.1007/s00520-025-10299-x)
Supplement: Supplementary file 3 — Supplementary file3 (DOCX 31 KB) [file 520_2025_10299_MOESM3_ESM.docx]

# Deceased (*n* = 4, 2.0%)

# Unable to contact (*n* = 33, 16.8%):

- Did not answer phone after maximum number of calls (*n* = 28)
- Address or phone number incorrect, or phone disconnected (*n* = 2)
- Other (*n* = 3)

# sent introductory mailing (*N* = 197)

# consented to participate (*n* = 112, 93.3% of # screened for eligibility)

# returned surveys (*n* = 99, 88.4% of # consented to participate)

# lost to follow-up (*n* = 5)

# withdrawn (*n* = 7)

# deceased (*n* = 1)

# surveys included in analyses (*n* = 99, 88.4% of # consented to participate)

# Not eligible (*n* = 8, 6.7% of # screened for eligibility):

- Declining health (*n* = 3)
- Cognitive impairment (*n* = 1)
- Vocal/auditory/visual impairment (*n* = 4)

# reached via phone (*n* = 160, 81.2%)

# screened for eligibility (*n* = 120, 75% of # reached via phone)

# Declined participation (*n* = 40, 25.0% of # reached via phone)^†^:

- Lack of interest (*n* = 20)
- No time (*n* = 6)
- Not feeling well (*n* = 4)
- Other (*n* = 12)

**Online Resource Figure 1.** Study recruitment flow chart.

^†^Respondents were allowed to choose more than one option for declining participation.
